# A reusable immobilization matrix for the biodegradation of phenol at 5000 mg/L

**DOI:** 10.1038/srep08628

**Published:** 2015-03-03

**Authors:** Najun Li, Jun Jiang, Dongyun Chen, Qingfeng Xu, Hua Li, Jianmei Lu

**Affiliations:** 1 State Key Laboratory of Treatments and Recycling for Organic Effluents by Adsorption in Petroleum and Chemical Industry, College of Chemistry, Chemical Engineering and Materials Science, Innovation Center of Suzhou Nano Science and Technology, Soochow University, Suzhou. 215123, China

## Abstract

Bacteria-mediated degradation of toxins has been reported as a practical technique for the innocuous removal of toxic organic compounds from water. Specifically, immobilized and pre-acclimatized *Pseudomonas putida* has been shown to clear low levels of contaminants (less than 2000 mg/L) from wastewater, wherein the bacteria consumes toxic aromatic compounds as the only source of carbon and energy. Here we report the preparation of a high-capacity composite adsorbent as an immobilization matrix for pre-acclimatized *P. putida* that is capable of complete degradation of 5000 mg/L of phenol within 80 hours. The composite adsorbent, a *n*-Butyl acrylate (BA)-resin evenly coated on polyester fiber (PF), can quickly reduce the phenol concentration to a level that is suitable for the immobilized bacteria to start the biodegradation process. Furthermore, the composite adsorbent (PF-BA) is regenerated during the biodegradation process without any additional manipulations, therefore it is reusable. As a whole, we provide a general strategy for more efficient biodegradation for phenol, which can be generalized to other water-soluble toxic organics removal for waste water treatment.

Phenol is a typical, toxic organic compound that is commonly used in petrochemical refineries, dye manufacturing and paper making factories. Phenol's water-solubility makes it a very important environmental pollutant. The toxicity of phenolic compounds limits the growth of aquatic life and also does harm to human health even at a low concentration^1^,^2^. Therefore, various technical processes such as solvent extraction^3^, biodegradation^4^,^5^, adsorption^6^,^7^ and chemical oxidation^8^,^9^ have been attempted to remove phenolic compounds from wastewater. As a practical, economical and environmentally-friendly technique, microbial degradation has attracted increasing attention in recent years. The popularity of this approach is mostly attributed to the development of immobilization technologies for microorganisms in wastewater treatment^10^,^11^,^12^,^13^,^14^. *P. putida* is known for its ability to degrade aromatic compounds, especially its high efficiency for phenol removal^15^,^16^.

However, *P. putida*'s low tolerance to toxic organic compounds restricted its application within the treatment of wastewater containing low concentrations (not exceeding 2000 mg/L) of contaminants, even after acclimation and immobilization of the bacteria^17^,^18^. More so, costly pretreatment processes such as adsorption and solvent extraction are required for treating sizeable industrial wastewater where the phenol concentration often reaches 3000–5000 mg/L.

Our previous work on acrylate resin-based adsorbents^19^ showed that the cross-linked BA-resin, polymerized from *n*-butyl acrylate monomers, had a high adsorption capacity for phenol with a great adsorption amount up to 1000 mg/g, and the adsorbents could rapidly reduce the phenol concentration from a high level to a relatively low level that the bacteria could tolerate (below 2000 mg/L). However, since the resin beads were prepared via traditional suspension polymerization method, they were too smooth and sticky to be used as the matrix for bacteria immobilization. To circumvent this problem, we used commercial polyester fiber (PF) flakes, which have porous reticular structure and hydrophilic surface, as a substrate for the BA-resin. After a self-modified two-phase interfacial solution polymerization, the BA-resin was evenly coated on the PF flakes. The resulting composite adsorbent (PF-BA) had a favorable adsorption capability for phenol, a reticular structure and a hydrophilic surface that could accommodate a large amount of bacterial cells. *P. putida* strain CICC 21906 was utilized for the biodegradation of phenol because of its high removal efficiency and low cost. The PF-BA-immobilized *P. putida* system was completed after acclimatization and immobilization (Fig. 1a). The reusable matrix allowed for the biodegradation of phenol at high concentration (up to 5000 mg/L) without any costly and laborious pretreatment. Our results indicate that the one-step adsorption-release-biodegradation synergetic process based on PF-BA-immobilized *P. putida* is much more efficient and convenient than the traditional multi-step method.

## Results

### Fabrication of the PF-BA composite adsorbent

**Figure 1:**
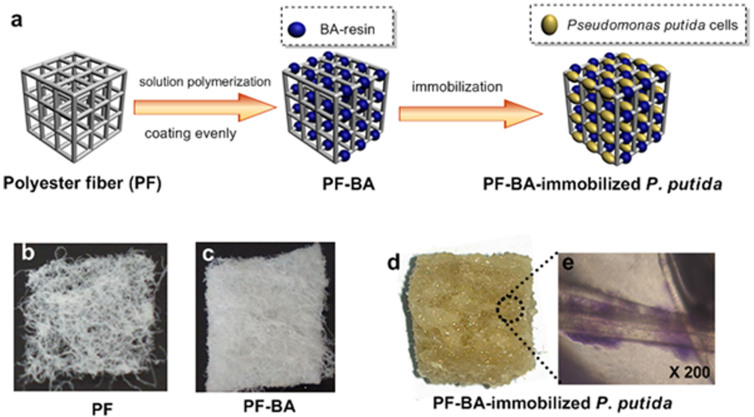
Characterization of the PF-BA composite adsorbent for immobilizing *P. putida*. (a) A schematic diagram of the fabrication process of the PF-BA composite adsorbent; (b) Optical images of polyester fiber (PF) flakes; (c) the composite adsorbent PF-BA; (d) PF-BA-immobilized *P. putida* and (e) microscopic images (200X) of the immobilized bacterial cells.

The hydrophilic surface and reticular structure (Fig. 1b) of the polyester fibers facilitated the loading of BA-resins, and resulted in an overall biocompatible surface for adsorbing bacterial cells. The composite adsorbent (PF-BA) was prepared by a modified solution polymerization at a water-methylbenzene interface. Upon polymerization, the BA-resin was evenly coated on the surface and interspaces of the polyester fiber (Fig. 1c). The resulting PF-BA had an average weight of 2729.41 g/m^2^ (10 mm-thick) and the average content of BA-resin in PF-BA was 83.59% by weight.

**Figure 2:**
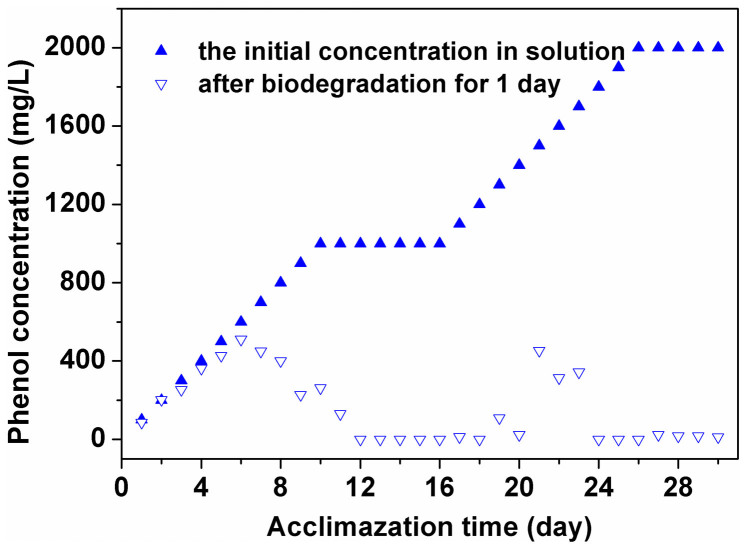
Acclimatization process of *P. putida.* (initial phenol concentration from 0 to 2000 mg/L, volume of minimal salts medium (MSM) solution = 50 mL, pH = 7 and T = 30°C).

Prior to immobilizing *P. putida* on the composite adsorbent, we acclimatized the bacteria to a phenol-based growth media (Fig. 2). The bacterial cells are centrifugal collected and add into the new MSM solution with the gradually increased phenol concentration per day. Since there was an “adaptive period” (from 1^st^ to 10^th^ day) during which *P. putida* uses phenol less efficiently as a sole carbon source, we slowly increased the phenol concentration as well as decreased the glucose concentration by 100 mg/L per day (the total amount of carbon source keeps on 1000 mg/L). We observed that *P. putida* began to adapt to phenol gradually. At 12 days post-acclimatization, *P. putida* could degrade phenol completely from an initial concentration of 1000 mg/L in the absence of glucose, which indicated that the newly acclimatized *P. putida* could use phenol as the only source of carbon and energy (supplementary Fig. 1). Keeping on the stable biodegradation capability for a few days (from 11^st^ to 16^th^ day), we continued to increase the phenol concentration in MSM solution. When increasing the phenol concentration (from 17^th^ to 26^th^ day, from 1100 mg/L to 2000 mg/L by 100 mg/L per day), the bacteria could achieve a higher level of biodegradability up to 2000 mg/L after the second “adaptive period” (from 27^th^ to 30^th^ day) although the cells began to decay after increasing the phenol concentration beyond 2000 mg/L. In other words, the free cells system of *P. putida* could be acclimatized to biodegrade phenol at a concentration below 2000 mg/L.

Upon immobilization on PF-BA, we observed that the acclimatized *P. putida* cells formed a thin biological membrane on the surface of the polyester fiber flakes (Fig. 1d) based on the affinity of bacteria to the porous matrix. The bacterial membrane was immobilized on PF-BA firmly and could not been washed away by running water. Upon Giemsa-staining, the living cells could be clearly observed under the microscope (Fig. 1e). We then collected the biofilm, dried it, and determined that the dry cell weight (DCW) was 89.64 mg/g.

### Biodegradation of phenol in high concentration phenolic water by PF-BA-immobilized *P. putida*

**Figure 3:**
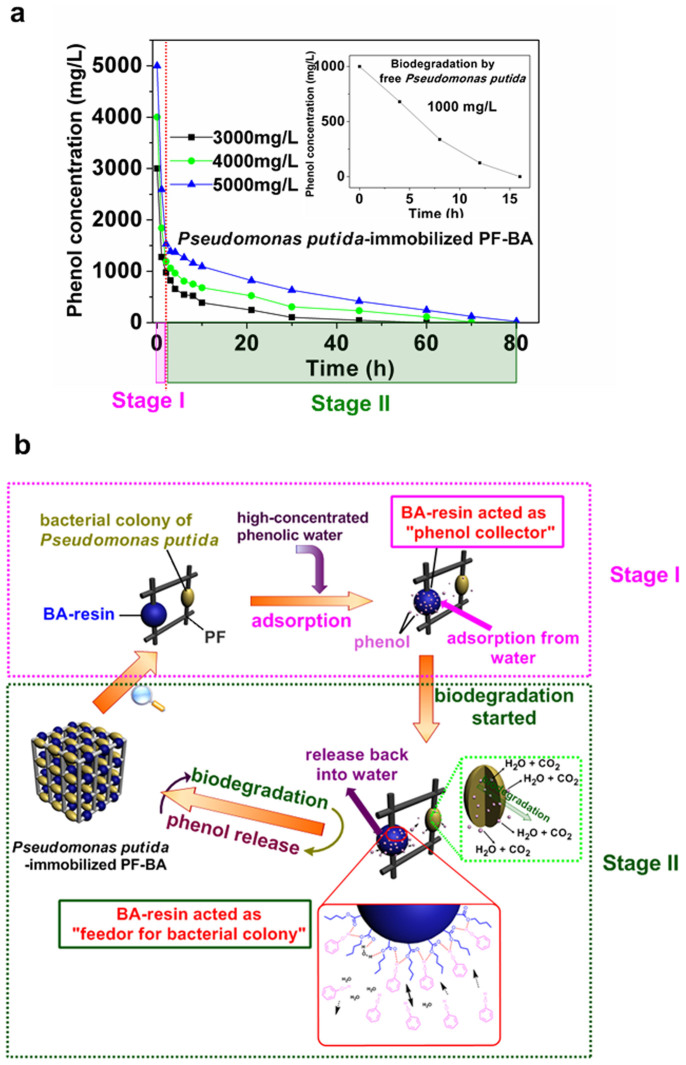
Biodegradation of phenol at high concentration by PF-BA-immobilized *P. putida.* (a) The experimental curves. (b) The schematic diagram of the process in (a).

The PF-BA-immobilized *P. putida* was directly placed into high concentration phenolic water (3000–5000 mg/L) for biodegradation of phenol. We observed that phenol was completely removed after 60, 70, and 80 hours from the initial concentration of 3000, 4000, and 5000 mg/L, respectively (Fig. 3a).The mechanism of this one-step phenol biodegradation process could be divided into two stages, which is illustrated by the schematic diagram in Fig. 3b. In **Stage I**, the concentration of phenol declined sharply to a low level (<1500 mg/L) within 1 hour, owing to PF-BA's high adsorption capacity for phenol. As a result, the circulating concentration of phenol that is exposed to the immobilized bacteria was less than 1500 mg/L, which is lower than tolerance threshold for phenol-acclimatized *P. putida* (2000 mg/L), and it is also much lower than the total available phenol in the system (3000–5000 mg/L). In **Stage II**, the biodegradation process happened gradually until phenol was completely removed from water within 80 hours.

**Figure 4:**
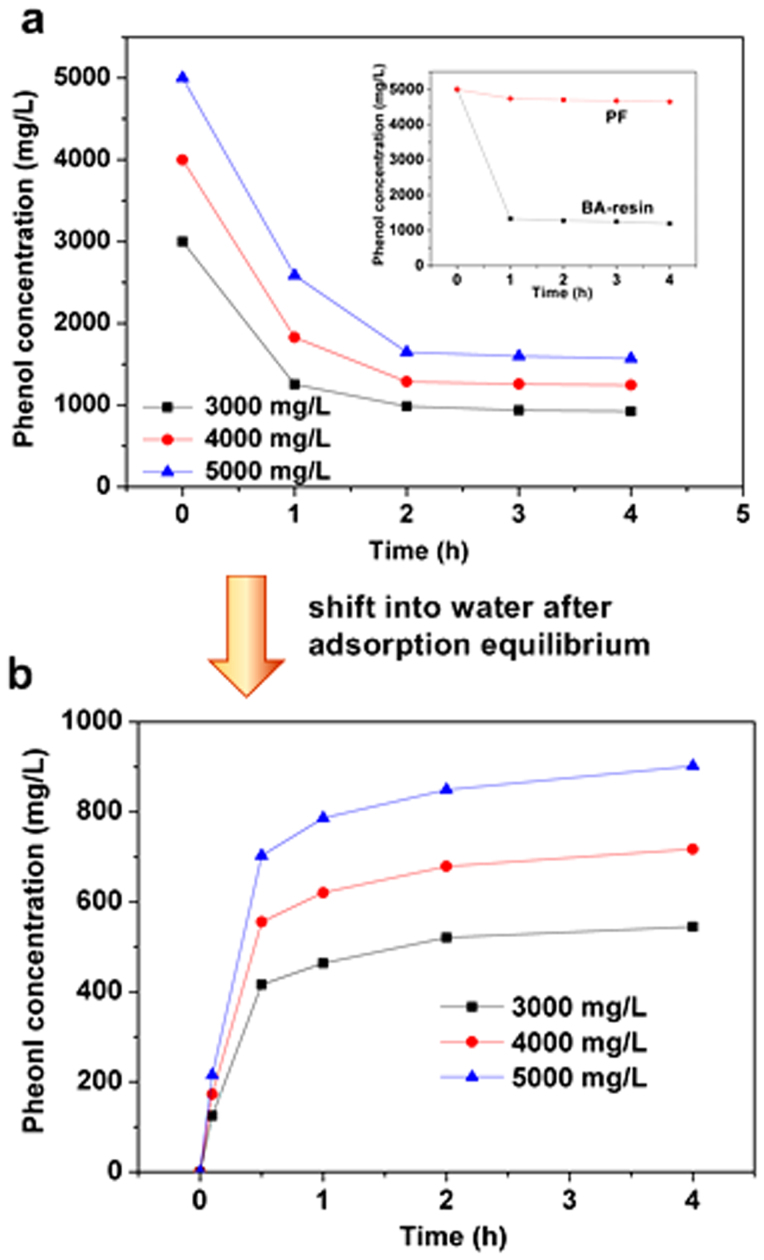
Adsorption characteristic of PF-BA. (a) Adsorption in highly-concentrated phenolic water (the inset shows the adsorption of its individual components, PF and BA-resin); (b) The slow release of phenol into water.

To understand the mechanism of **Stage I**, we investigated the adsorption of phenol by PF-BA without immobilized *P. putida* at phenol concentrations (3000–5000 mg/L) that are much higher than the maximum concentration (2000 mg/L) tolerated by the bacteria and which closely resemble conditions in industrial wastewater (Fig. 4a). We observed that most phenol molecules were adsorbed from water, and the phenol concentration declined dramatically within 1 hour, just as we observed in **Stage I** of PF-BA-immobilized *P. putida* (Fig. 3a). However, the adsorption equilibrium of PF-BA occurred at different initial concentrations for the two systems.

Without the *P. putida*-mediated biodegradation of phenol, the adsorption equilibrium of PF-BA was maintained at a low concentration, with an average removal ratio of around 70% (Fig. 4a). In addition, the adsorption of phenol by polyester fiber (PF) or BA-resin alone was investigated (Fig. 4a inset). The commercial PF itself has little adsorption capacity of phenol with a low removal rate (less than 10%), but the BA-resin beads reached a favorable removal rate of 76% within 1 hour. So the adsorption property of BA-resin is retained in the composite adsorbent PF-BA. Although PF-BA could not remove phenol 100% from water, its high adsorption capacity (with a great adsorption amount up to 1000 mg/g for phenol) made it be a suitable matrix for *P. putida* immobilization in high concentration phenolic water to protect the bacterial cells from toxic condition in time. Therefore, the bacterial cells of *P. putida* immobilized on PF-BA can tolerate much higher phenol concentration (3000–5000 mg/L) than those on other common matrix such as activated pumice particles (1000 mg/L)^20^ and polyvinyl alcohol gel particles (300 mg/L)^15^.

The adsorption isotherm of phenol by PF-BA was investigated with the frequently employed models, Langmuir and Freundlich isotherm models^21^ (supplementary Fig. 2 and Table 1). The Langmuir isotherm model assumes that the adsorption process occurred at the monolayer of the surface and that there is no interaction between the adsorbed solute molecules and those that are free in the solution. The Freundlich isotherm, however, supposes that the adsorption takes place between multilayers. Our results indicate that the Freundlich isotherm model better describes the adsorption equilibrium of phenol by PF-BA. That was to say, that multilayer adsorption plays a major role in the adsorption process. The model also shows that the adsorption of phenol by PF-BA could be divided into two stages, one rapid adsorption process with a sharply reduced concentration and another slow equilibrium state. It took a relatively longer time (about 1 hour) to reach anequilibrium state with a PF-BA system as compared to other porous adsorbents such as active carbon^22^ for about several minutes.

We found that the composite adsorbent (PF-BA) could release the phenol back into water slowly, which is advantageous to the gradual biodegradation by *P. putida*. This feature was verified by a phenol-release experiment (Fig. 4b). After the adsorption of phenol reached equilibrium, the phenol solution was replaced by the same volume of distilled water. By so doing, the hydrogen bonding interactions between the resin surface and phenol molecules were weakened by the competition from more water molecules. The result was an equilibrium shift, such that some of the phenol molecules that had initially adsorbed to the BA-resin were gradually released back to the solution until a new equilibrium was achieved. Since the maximum phenol concentration at equilibrium was no more than 1000 mg/L, the bacterial colonies of *P. putida* were shielded from an otherwise toxic concentration of phenol within the confines of the biodegradation well. In fact, the acclimatized bacteria maintained a high biodegradation activity as soon as the phenol concentration in solution decreased below their upper limit (2000 mg/L). Therefore, as *P. putida* degraded the soluble phenol, more and more phenol molecules were released from the saturated PF-BA matrix back into the aqueous solution. Accordingly, the adsorption equilibrium above was constantly adjusted until phenol was removed completely (0 mg/L in water). This is in a stark contrast to the simple adsorption process of PF-BA composite adsorbent, which maintained a fixed equilibrium concentration.

**Figure 5:**
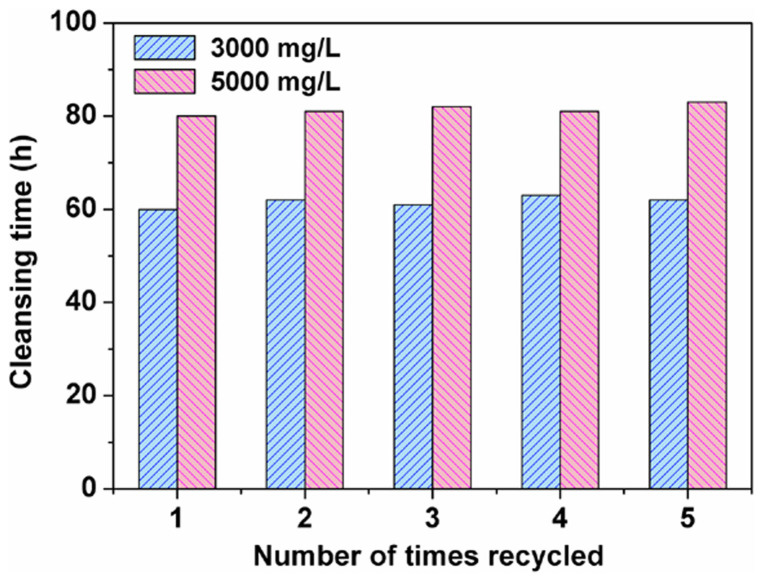
Efficiency of reusable PF-BA-immobilized *P. putida* for clearing high concentrations of phenol in water.

It should be mentioned that the regeneration of PF-BA happened concomitantly to the biodegradation process; such that as soon as the biodegradation of phenol was complete, the matrix could be reused for subsequent treatments of phenolic water without any further processing. Even after several cycles, the PF-BA-immobilized *P. putida* system maintained the same processing efficiency as the first cycle (Fig. 5).

## Discussion

In this work, we introduced an immobilization matrix that stabilizes *P. putida* and mediates the efficient step-wise adsorption and biodegradation of phenol.

Traditional matrices for bacterial immobilization are porous inorganic materials with macropores that have low adsorption capacity. In contrast, we generated a high capacity, biocompatible adsorbent material. On its own, the cross-linked BA-resin had a high adsorption capacity for phenol; however, since the resin beads were prepared via traditional suspension polymerization method, they were too smooth and sticky to be directly used as the matrix of bacteria immobilization. Instead, we employed commercial polyester fiber (PF) flakes, which have porous reticular structures and a hydrophilic surface, as the substrate for the BA-resin. The acclimatized bacteria were then immobilized on to the composite adsorbent PF-BA. It is worth mentioning that the bacteria were immobilized in the meshes of the reticular polyester fiber flakes, and not the smooth surface of the BA-resin. The *P. putida* colonies and BA-resins were alternately distributed over the reticular fiber flakes to facilitate mass transfer and diffusion of phenol after adsorption from water.

In all, the immobilized bacteria and PF-BA acted synergistically: The high-capacity resin rapidly adsorbed phenol from water and shielded the bacteria from the toxicity of high levels of phenol; as the bacteria degraded the low-level (1000 mg/L) of phenol in circulation, more and more phenol was released until all of the phenol was completely degraded.

The process of treating phenolic water at high concentrations can be divided into two stages: 1) an adsorption stage, and 2) a release-and-biodegradation stage. At the beginning of the adsorption process, the adsorption rate of phenol was very fast, given the abundance of vacant adsorption sites on the surface of BA-resin. Adsorption is mediated by the hydrogen bonding interaction between the hydroxyl groups on phenol molecules and the carbonyl groups on the surface of BA-resin beads. Upon saturation, the phenol adsorption is changed into multilayer adsorption from monolayer adsorption when the vacant adsorption sites on the surface of BA-resin are filled up. The hydrogen bonding between the multilayer of phenol molecules and the surface of BA-resin is much weaker than that between the monolayer of phenol molecules and the surface of BA-resin, and it takes a relatively long time for the phenol molecules on the monolayer to diffuse inside the BA-resin. In addition, the competition between water and BA-resin to phenol molecule brings about desorption of phenol from PF-BA. So a dynamic equilibrium is formed between the adsorption and desorption of phenol. Though it took a relatively long time for the system to reach equilibrium (Fig. 3b), the ability of PF-BA to adsorb and release phenol back into solution made it possible for *P. putida* to clear highly concentrated phenol solutions (3000–5000 mg/L) in the subsequent biodegradation step. With phenol release from PF-BA and its active biodegradation by the immobilized *P. putida*, the adsorption equilibrium was broken constantly until the phenol was removed completely from the system. Under the synergy of adsorption-release-biodegradation process, the highly concentrated phenolic water could be cleansed within 60, 70 and 80 hours from the initial concentrations of 3000, 4000 and 5000 mg/L, respectively.

In conclusion, a composite adsorbent was fabricated by coating a high-capacity BA-resin on to polyester fiber (PF) flakes. The resulting matrix was then used to immobilize acclimatized *P. putida* for phenol degradation at the high concentrations of 3000 to 5000 mg/L. The PF-BA-immobilized *P. putida* system could be reused multiple times to cleanse high concentration phenolic water (5000 mg/L) without any pretreatment or regeneration procedure. Taken together, we have generated a new strategy for one-step biodegradation of highly concentrated phenolic water based on the synergy of adsorption and biodegradation that is conferred by BA-resin and immobilized *P. putida*.

## Methods

### Reagents

*n*-Butyl acrylate (*n*-BA), methyl methacrylate (MMA), 2-ethylhexyl acrylate (2-EHA), lauryl methacrylate (LMA), benzoyl peroxide (BPO), ethylene glycol dimethacrylate (EDMA, cross-linking agent), phenol, inorganic salts and analytical reagent grade were commercially obtained from Sinopharm Chemical Reagent Co., Ltd and used as received without further modification. Double-distilled water was filtered through a Millipore membrane filter before being used. The fiber substrate (polyester fiber, 505 g/m^2^ in weight and 2 cm in thickness) was purchased from Shanghai Yanpai Filter-cloth Co., Ltd. A pure strain of *P. putida* (CICC 21906) was purchased from China Center of Industrial Culture Collection. Tryptone and Yeast extract were purchased from Suzhou Biogene Biotechnology Co., Ltd.

### Fabrication of the composite adsorbent (PF-BA) and immobilization of *P. putida*

PF-BA was obtained after the cross-linked polymerization of *n*-butyl acrylate monomers on the polyester fiber (PF), using EDMA as the crosslinking agent. First, 2.0 g BPO and 1.0 g EDMA were dissolved in a mixture of 200 ml *n*-BA and 200 ml methylbenzene in an enamel vessel. Next, a piece of polyester fiber sheet (300 × 200 mm^2^, 10 mm-thickness) was dipped into the above organic solution mixed with 800 ml H_2_O so that the monomers could polymerize on the polyester fiber sheet evenly during the polymerization process in a 95°C water bath for 5 h. After washing with ethanol and distilled water, PF-BA was dried at room temperature and cut into small pieces (5 × 5 × 5 mm^3^) for later use.

Luria Bertani (LB) liquid medium (Tryptone, 10 g/L; Yeast extract, 5 g/L; NaCl, 10 g/L), minimal salts medium (MSM) solution and all the apparatus were autoclaved at 121°C for 15 min, and the glucose and phenol were separately sterilized by membrane filter (0.22 μm). A pure strain of *P. putida* (CICC 21906) was purchased from CICC (China Center of Industrial Culture Collection), and activated in liquid LB medium to obtain a certain phenol-degradation activity and biomass. *P. putida* was then transferred into a 50 ml MSM solution containing 1000 mg/L glucose (a commonly used organic carbon source) and incubated at 30°C, 200 rpm.

The acclimatization process was achieved by gradually increasing the phenol concentration by 100 mg/L per day until it reached 2000 mg/L (the upper limit) while simultaneously decreasing the glucose concentration by 100 mg/L per day until it reached 0 mg/L in the MSM solution. At this stage, the bacteria were considered fully acclimatized to phenol as a sole carbon and energy source. The acclimatized bacteria were then used for phenol biodegradation and immobilization studies.

The immobilization process was carried out in a 250 ml Erlenmeyer flask, plating 50 ml suspension of acclimatized *P. putida* (in an MSM solution containing 1000 mg/L of phenol) in a thermostatic incubator. Next, 7.0 g of PF-BA was added in the suspension and kept shaking in the constant temperature oscillator at 30°C for several days. The loading amount of *P. putida* cells was estimated by determining the difference of the dry cell weight (DCW) before and after immobilization. The DCW was calculated from OD_600_ by a standard curve.

To determine the optimal conditions of the biodegradation of phenol by *P. putida*, batch experiments were carried out at different pH (2–12) and temperatures (25–45°C) with identical initial phenol concentration of 1000 mg/L. (Subsequent experiments were perofrmed atthe optimal conditions of 30°C, pH = 7.0).

### Batch experiments

All batch experiments were carried out in 250 ml Erlenmeyer flasks (50 ml solution), shaking in a constant temperature oscillator (SHA-CA).The adsorption amount of phenol (Q) was calculated by formula (1)^23^:
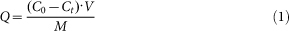
Where *C_0_* is the initial phenol concentration (mg/L), *C_t_* is the residual phenol concentration at time *t* (mg/L), *V* is the volume of the solution (L) and *M* is the dosage of the composite adsorbent with or without immobilized bacteria (g).

### Analytical methods

Phenol concentration was determined against a calibration curve of standard phenol solutions of known concentration by high performance liquid chromatography (HPLC, Waters 2489) using an Accurasil C18 column (4.6 mm × 150 mm inner diameter, 5 μm particle size) with a mobile phase of methyl alcohol/water (3/7, v/v) pumped at 1 ml/min. The sample was filtered through a syringe filter having a pore size of 0.22 μm before being injected into the HPLC. The optical density (OD) analysis was used to indicate cell density using a UV-Vis spectrophotometer at 600 nm. The OD_600_ value could be converted to dry cell weight using a standard curve. The pH value was monitored with a HANNA pH meter. All the experiments were carried out in triplicate and the average values were reported.

## Author Contributions

J.Lu conceived the project; N.L. designed and performed the experiments; J.J., D.C., H.L. and Q.X. contributed to data analysis; N.L. wrote the manuscript. All the authors reviewed the manuscript.

## Supplementary Material

Supplementary Information A reusable immobilization matrix for the biodegradation of phenol at 5000 mg/L
