# Supplementary material for: A reusable immobilization matrix for the biodegradation of phenol at 5000 mg/L
Source: Sci Rep. 2015 Mar 3;5:8628. doi: 10.1038/srep08628 (PMC4347024; doi:10.1038/srep08628)
Supplement: Supplementary Information — A reusable immobilization matrix for the biodegradation of phenol at 5000 mg/L [file srep08628-s1.pdf]

A reusable immobilization matrix for the biodegradation of phenol at 5000 mg/L

Najun Li, Jun Jiang, Dongyun Chen, Qingfeng Xu, Hua Li, Jianmei Lu*

State KeyLaboratory of Treatments and Recycling for Organic Effluents by Adsorption in Petroleum and Chemical Industry, College of Chemistry, Chemical Engineering and Materials Science, Innovation Center of Suzhou Nano Science and Technology, Soochow University, Suzhou, 215123 China

*Correspondence to: J. M. Lu (E-mail: lujm@suda.edu.cn), Tel/Fax: 86 512 65880367.

Supplementary Information

Biodegradation of phenol by free *Pseudomonas putida*

To determine the rate of phenol biodegradation by *P. putida*cells (in the absence of the PF-BA matrix), we challenged the bacteria with 1000 mg/L of phenol in MSM solution, pH 7.0 at 30˚Cfor 16h. In that time, the phenol concentration decreased almost linearly withtime and cell density (as measured from OD_{600}) **(Fig. S1a)**. The increasing amount of DCW was calculated to be 8.25 mg against thestandard curve (**Fig. S1b**).

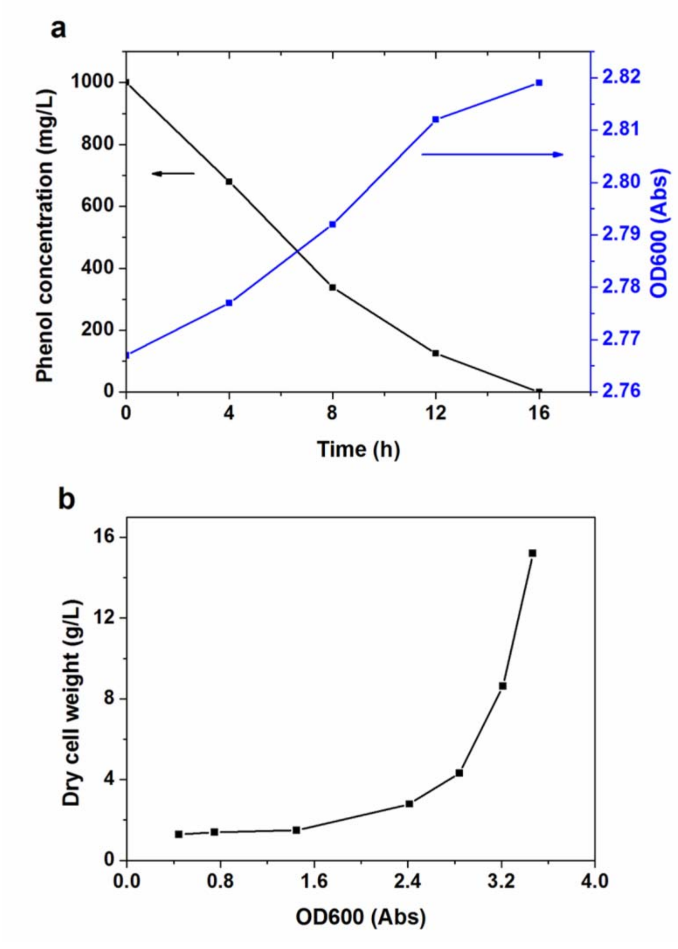

Fig. S1 (a) Biodegradation curve and OD_{600}offree*P. putida*(initial phenol concentration=1000 mg/L, volume of phenol solution=50 mL, pH=7.0 and T=30 ˚C) and (b)the relative conversion curve of DCW and OD_{600}.

To determine the optimal conditions of the biodegradation of phenol by *Pseudomonas putida*cells, batch experiments were carried out at different pH (2-12) and temperatures (25-45˚C) respectively with a identical initial phenol concentration of 1000 mg/L.The results shown in **Fig. S2** indicated that it had a favourable

biodegradation rate at the optimized temperature of 30˚C and the optimum pH value near neutrality. So all the experiments were perofrmed under this optimal condition (30˚C, pH=7.0).

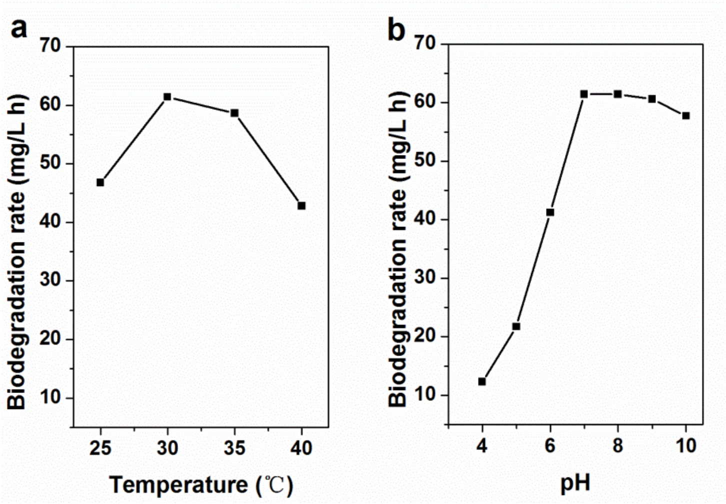

Fig. S2 Effect of temperature (a) and pH (b) on biodegradation rate of phenol (initial phenol concentration=1000 mg/L, volume of phenol solution=50 mL)

Adsorption isotherm of phenol by PF-BA

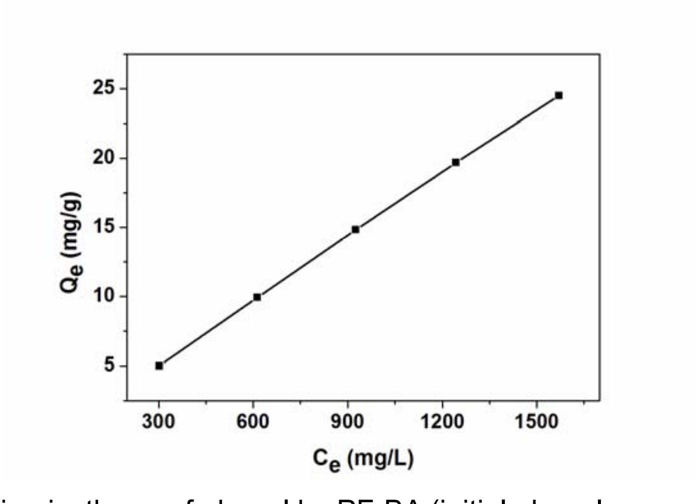

Fig. S3 Adsorption isotherm of phenol by PF-BA (initial phenol concentration=1000, 2000, 3000, 4000, 5000 mg/L, weight of PF-BA= 7.0 g, $V_{phenol\ solution}$=50 mL, pH=7.0 and T=30 ˚C

According to the adsorption equilibrium data (**Fig. S3**), the value of Q_e (the adsorption amount of phenol) was found to increase linearly with increasing C_e (the equilibrium concentration) at30 ˚C. That was to say, the initial phenol concentration had little effect on the adsorption capability of PF-BA, and that the composite

adsorbentcould be used directly at high phenol concentration (5000 mg/L) to meet the industrial requirements.

In order to further evaluate the adsorption of phenol by PF-BA, the most frequently employed models, Langmuir and Freundlich isotherm models were used to describe the adsorption process. The Langmuir isotherm model is based on the supposition that the adsorption process happened at the monolayer of the surface, and that there is no interaction between the solute molecules adsorbed on the surface and those that are free in the solution. The linear form of the Langmuir isotherm model can be written as:

$$\frac{C_e}{q_e} = \frac{C_e}{Q_m} + \frac{1}{K_L \cdot Q_m} \quad (1)$$

Where q_e is the adsorption amount of phenol at equilibrium (mg/g), Q_m is the theoretical monolayer capacity (mg/g), C_e is the residual phenol concentration at equilibrium (mg/L) and K_L is the constant. The Freundlich isotherm supposes that the adsorption takes place between multilayers. The logarithmic linear form of the Freundlich isotherm model is given as:

$$\ln Q_e = \ln K_F + (1/n)\ln C_e \quad (2)$$

Where K_F is the Freundlich isotherm constant (L/g) reflecting the adsorption capacity and 1/n is the value of the adsorption intensity.

Table S1 Isotherm parameters for phenol adsorption by PF-BA

T/K	Freundlich model			Langmuir model		
	$\ln Q_e = \ln K_F + (1/n)\ln C_e$			$C_e/Q_e = C_e/Q_m + 1/(K_L Q_m)$		
	K_F	n	R^2	Q_m	K_L	R^2
303K	0.019726	1.03213	0.9999	392.1569	0.000042	0.9880

The values of isotherm constants and correlation coefficient (R^2) are listed in **Table S1**. The R^2 of Freundlich isotherm model (0.9999) was a little higher than that of the Langmuir of isotherm model (0.9880).
